# Efficient derivation of extraembryonic endoderm stem cell lines from mouse postimplantation embryos

**DOI:** 10.1038/srep39457

**Published:** 2016-12-19

**Authors:** Jiangwei Lin, Mona Khan, Bolek Zapiec, Peter Mombaerts

**Affiliations:** 1 Max Planck Research Unit for Neurogenetics, Max-von-Laue-Strasse 4, 60438 Frankfurt, Germany

## Abstract

Various types of stem cell lines have been derived from preimplantation or postimplantation mouse embryos: embryonic stem cell lines, epiblast stem cell lines, and trophoblast stem cell lines. It is not known if extraembryonic endoderm stem (XEN) cell lines can be derived from postimplantation mouse embryos. Here, we report the derivation of 77 XEN cell lines from 85 postimplantation embryos at embryonic day E5.5 or E6.5, in parallel to the derivation of 41 XEN lines from 69 preimplantation embryos at the blastocyst stage. We attain a success rate of 100% of XEN cell line derivation with our E5.5 whole-embryo and E6.5 disaggregated-embryo methods. Immunofluorescence and NanoString gene expression analyses indicate that the XEN cell lines that we derived from postimplantation embryos (post-XEN) are very similar to the XEN cell lines that we derived from preimplantation embryos (pre-XEN) using a conventional method. After injection into blastocysts, post-XEN cells contribute to extraembryonic endoderm in chimeras at E6.5 and E7.5.

Mouse preimplantation embryonic development culminates in the blastocyst stage. A blastocyst consists of three cell lineages: epiblast, trophectoderm, and primitive endoderm (PrE). The epiblast develops into most of the embryo proper, the amnion, and the extraembryonic mesoderm of the yolk sac; the trophectoderm gives rise ultimately to the fetal portion of the placenta; and the primitive endoderm forms the two extraembryonic endoderm lineages – the visceral endoderm (VE) and the parietal endoderm (PE) of the yolk sac^1^,^2^. The extraembryonic endoderm provides nutritive support to the embryo, and is required for several inductive events such as anterior patterning and formation of endothelial cells and blood islands^3^,^4^,^5^.

Stem cell lines have been derived from these three cell lineages^6^. Embryonic stem (ES) cell lines from epiblast were first reported in the 1980 s (refs 7 and 8), trophoblast stem (TS) cell lines from trophectoderm in the 1990 s (ref. 9), and extraembryonic endoderm stem (XEN) cell lines from PrE in the 2000 s (ref. 10). The conventional source of these cell lines is the blastocyst stage embryo. TS cell lines can also be derived from postimplantation embryos^9^,^11^,^12^. Moreover, mouse epiblast stem cell (EpiSC) lines, which resemble ES cell lines of human, can be derived from preimplantation embryos^13^ and postimplantation embryos^14^,^15^, and can be reverted to ES cells^16^. XEN cell lines are useful for the investigation of signaling pathways of cells of the extraembryonic endoderm lineages, and represent an *in vitro* model to identify patterning activities of the extraembryonic endoderm such as factors involved in cardiac induction^17^,^18^. Mouse fibroblasts pass via a XEN-like state on their way to induced pluripotent stem (iPS) cells by chemical reprogramming^19^.

There are three methods to derive mouse XEN cell lines^20^. The first method entails the direct derivation of XEN cell lines from blastocysts^10^. The second method involves the conversion of an existing ES cell line to a XEN or XEN-like cell line, either by forced expression of a transcription factor gene encoding *Gata4* or *Gata6* (refs 21, 22, 23) or *Sox17* (refs 24 and 25), or by chemical modification of the culture medium such as by addition of retinoic acid and activin A^26^. A third, more recently reported method, derives induced XEN cells (iXEN) by reprogramming fibroblasts with the classical iPS reprogramming factors *Oct4, Sox2, Klf4*, and *Myc*; colonies from which iXEN cells can be derived, arise in parallel to iPS cells^27^.

Here, we show that XEN cell lines can be derived with very high efficiency from postimplantation embryos at E5.5 or E6.5, either from whole or disaggregated embryos. These so-called post-XEN cell lines are very similar to the pre-XEN cell lines that we derived directly from preimplantation embryos.

## Results

### Derivation of pre-XEN cell lines from blastocysts

To distinguish unambiguously the XEN cell lines that were derived from preimplantation embryos from the XEN cell lines that were derived from postimplantation embyros, we refer to these cell lines operationally as pre-XEN and post-XEN cell lines, respectively.

**Figure 1:**
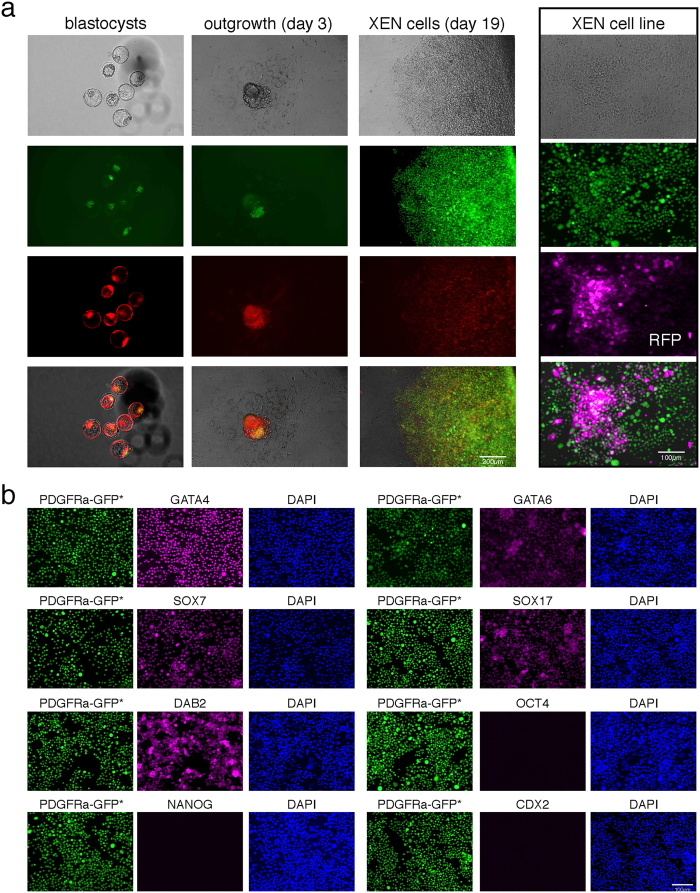
Derivation of pre-XEN cell lines from blastocysts. (**a**) PDGFRa-GFP × CAG::mRFP1 blastocysts and pre-XEN cell line X47. From left to right: blastocysts, outgrowth of blastocyst after 3 days in culture, cells after disaggregation of the outgrowth and culture for 19 days, and established pre-XEN cell line on day 38. From top to bottom, first three columns: bright-field image, intrinsic green fluorescence of GFP, intrinsic red fluorescence of RFP, and combined green and red fluorescence/bright-field image. From top to bottom, right-most column: bright-field image, intrinsic green fluorescence of GFP, immunofluorescence for RFP, and combined green fluorescence and immunofluorescence/bright-field image. (**b**) Fluorescence analysis of pre-XEN cell line X42. Shown are eight pairwise combinations of intrinsic (indicated with an asterisk after GFP) green fluorescence from the gene-targeted *PDGFRa* locus and immunofluorescence (magenta), together with DAPI (blue). Cells are immunoreactive for XEN markers GATA4, GATA6, SOX7, SOX17, and DAB2. But cells are negative for ES cell markers OCT4 and NANOG, and for TS cell marker CDX2.

We first derived a set of conventional pre-XEN cell lines from blastocysts, in order to set up conditions in our laboratory and to provide a comparison for post-XEN cell lines. We collected 63 E1.5 embryos from three types of natural matings: from three heterozygous PDGFRa-GFP females^28^ mated with homozygous CAG::mRFP1 males^29^, two B6D2F1 females mated with hemizygous D4/XEGFP males^30^, and two homozygous D4/XEGFP females mated with DBA/2 N males (Table 1). PDGFRa is a XEN-cell marker^26^,^31^,^32^; CAG::mRFP1 is a transgene that expresses ubiquitously the red fluorescent protein; D4/XEGFP is a transgene integrated on the X-chromosome, and is not expressed from the inactive X-chromosome, enabling the future study of X-chromosome inactivation in XEN cell lines. We cultured the embryos in KSOM medium until the blastocyst stage, and then removed the zona pellucida using acid Tyrode solution. We transferred each blastocyst separately into a well of a 4 well-dish coated with 0.1% gelatin and covered with mouse embryonic fibroblasts (MEF), and switched to ES cell medium supplemented with leukemia inhibitory factor (LIF)^20^. All blastocyst cells including PrE cells express RFP from the transgene (Fig. 1a). After 3 days in culture, blastocysts started to form outgrowths (Fig. 1a), which we disaggregated on day 5. Cell line X47 was still in the process of becoming established (and was still displaying intrinsic red fluorescence) after 19 days in culture (Fig. 1a). We obtained a stable pre-XEN cell line X47 after 38 days of culture (Fig. 1a). The CAG promoter does not appear to generate sufficient mRFP1 to enable us to detect the intrinsic red fluorescence in XEN cell lines, similar to what has been reported for CAG::H2B-GFP and CAG::YFP strains^26^. We thus derived, using the conventional method^20^ with ES cell medium and LIF, a total of 36 pre-XEN cell lines from 63 blastocysts, at a 57% success rate (Table 1).

Next, we isolated by immunosurgery^33^ the inner cell mass (ICM) of blastocysts from a natural mating between an R26-tauGFP41 female and a Sox17-Cre male^34^. With Sox17 a XEN-cell marker, and R26-tauGFP41 a Cre reporter strain, this cross marks permanently cells that have been derived from Sox17-expressing cells. We obtained 6 ICMs from 6 blastocysts, which we had collected at E2.5 as morulae and cultured overnight in KSOM medium. We transferred each ICM separately into a well of a 4-well dish coated with gelatin and covered with MEF, and cultured the ICM outgrowths in ES medium with LIF. Seven days later we replaced the ES medium by TS medium with 25 ng/ml FGF4 and 1 μg/ml heparin (referred to as F4H) without passaging the cells. We changed the medium every two days, and on day 14 we passaged the cells to 12-well dishes. We thus established five XEN cell lines from six ICMs, at a success rate of 83% (Table 1).

Figure 1b shows immunofluorescence staining of pre-XEN cell line X42, from the cross PDGFRa-GFP × CAG::mRFP1. Cells also display intrinsic green fluorescence of GFP produced from the gene-targeted *PDGFRa-GFP* locus (indicated with the asterisk PDGFRa-GFP*). We find that this and other pre-XEN cell lines are immunoreactive for XEN cell markers GATA4, GATA6, SOX7, SOX17, and DAB2, but negative for ES cell markers OCT4 and NANOG, and negative for TS cell marker CDX2.

### Derivation of post-XEN cell lines from whole E6.5 embryos

**Figure 2:**
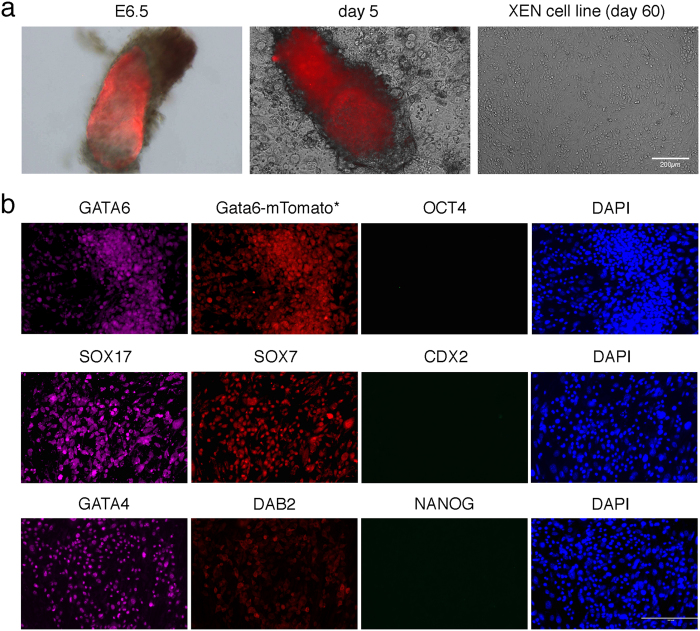
Derivation of post-XEN cell lines from whole E6.5 embryos. (**a**) Gata6-mTomato × Cdx2-GFP embryo and post-XEN cell line X-E6.5-82278-4. From left to right: whole E6.5 embryo, large outgrowth of embryo after 5 days in culture, and established post-XEN cell line on day 60. The embryo and the outgrowth display intrinsic red fluorescence of mTomato, but the expression of mTomato in the post-XEN cell line is below the detection level of intrinsic red fluorescence from mTomato. (**b**) Immunofluorescence analysis of post-XEN cell line X-E6.5-82278-4. Expression of mTomato from the *Gata6* promoter in the transgene is detectable with an antibody for RFP (first row, second column). Cells are immunoreactive (magenta) for XEN markers GATA4, GATA6, SOX7, SOX17, and DAB2. But cells are negative for ES cell markers OCT4 and NANOG, and for TS cell marker CDX2. Right-most column shows DAPI (blue).

Next we collected E6.5 postimplantation embryos from three types of natural matings: two heterozygous Xist1loxGFP females^35^ mated with a wild-type DBA/2 N male, two heterozygous ROSA26-STOP-taulacZ females mated with a heterozygous Sox17-Cre male^34^, and one hemizygous Gata6-mTomato female^36^ mated with a homozygous Cdx2-GFP male^37^ (Table 1). Xist1loxGFP is a GFP-containing targeted mutation in the *Xist* locus on the X-chromosome; Sox17 and Gata6 are XEN-cell markers; and Cdx2 is a marker for trophoblast stem cells. We removed the ectoplacental cone of the embryos as much as possible, and transferred each embryo separately into a well of 4-well dish coated with 0.1% gelatin and covered with MEF in TS cell medium including 25 ng/ml FGF4 and 1 μg/ml heparin (referred to as F4H). One day later, the embryos had attached to the surface and started to form an outgrowth. The embryos had formed a large outgrowth after 5 days. We used TrypLE Express to disaggregate the outgrowths and passaged cells into a well of a 4-well dish. After cells reached 70–80% confluency, they were passaged into a well of a 12-well dish. After they reached 70–80% confluency again, cells were passaged into a well of a 6-well dish, and we then obtained stable post-XEN cell lines. The intrinsic red fluorescence of mTomato produced from the *Gata6* promoter in the transgene was sufficiently high to detect it in the whole embryo and outgrowth, but not in the established post-XEN cell line at day 60 (Fig. 2a). We thus derived, using the whole-embryo method, a total of 30 post-XEN cell lines from 38 E6.5 embryos, at a 79% success rate (Table 1).

We found that mTomato expressed from the *Gata6* transgenic promoter in these cells can be detected by immunofluorescence with antibodies against RFP, together with GATA6 expressed from the endogenous *Gata6* locus with antibodies against GATA6 (Fig. 2b). As was the case for our pre-XEN cell lines, our post-XEN cell lines are positive for XEN cell markers GATA4, GATA6, SOX7, SOX17, and DAB2 but negative for ES cell markers OCT4 and NANOG, and negative for TS cell marker CDX2.

### Derivation of post-XEN cell lines from disaggregated E6.5 embryos

**Figure 3:**
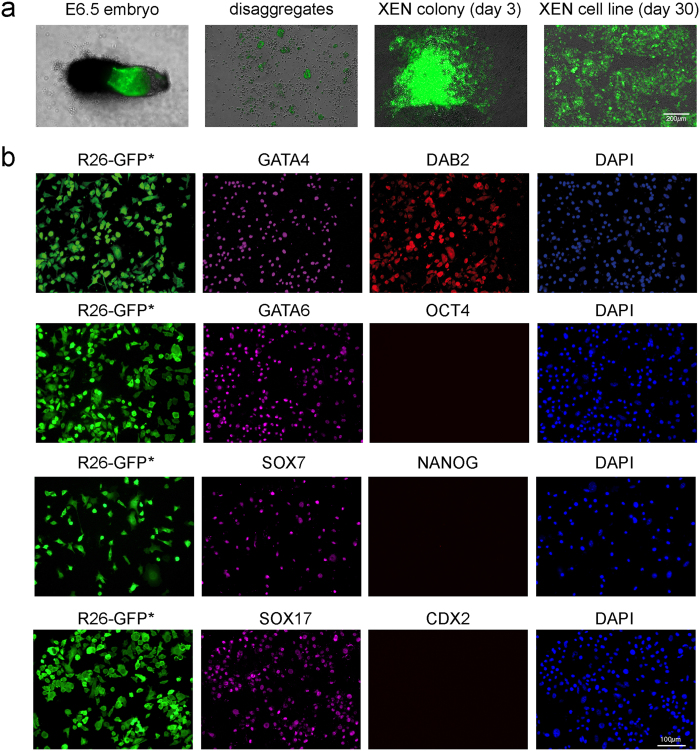
Derivation of post-XEN cell lines from disaggregates of E6.5 embryos. (**a**) R26-tauGFP41 × Sox17-Cre E6.5 embryo and post-XEN cell line X-E6.5-Z0617-2. From left to right: whole E6.5 embryo, disaggregated embryo, XEN-like colony expressing GFP on day 3 of culture, and established post-XEN cell line on day 30. Intrinsic green fluorescence of GFP. (**b**) Fluorescence analysis of post-XEN cell line X-E6.5-Z0617-5. First column: intrinsic (indicated with an asterisk after GFP) green fluorescence of GFP expressed from the *ROSA26* locus after activation by Cre recombinase that is expressed from the gene-targeted *Sox17* locus. Second column: cells are immunoreactive (magenta) for XEN markers GATA4, GATA6, SOX7, and SOX17. Third column: cells are immunoreactive for XEN marker DAB2, but negative for ES cell markers OCT4 and NANOG, and negative for TS cell marker CDX2. Fourth column: DAPI (blue).

During the process of post-XEN cell line derivation from whole E6.5 embryos, we observed that some embryos had difficulty forming large outgrowths. We reasoned that some unidentified cell types in these embryos may inhibit XEN cell growth. We therefore proceeded to derive post-XEN cell lines from disaggregates of these embryos. We isolated 30 E6.5 embryos from three types of natural matings: a homozygous R26-tauGFP41 female with a heterozygous Sox17-Cre male^36^, a heterozygous ROSA26-STOP-taulacZ female with a heterozygous Sox17-Cre male, and a wild-type CD1 female with a heterozygous PDGFRa-GFP male^28^ (Table 1). We prepared disaggregates (Fig. 3a) by exposing the embryos to collagenase and deoxyribonuclease followed by TrypLE Express, and then gently pipetting the embryos in a glass pipette with a diameter of 50–60 μm. We plated the cell suspension, which consisted of a mixture of single cells and clumps of cells, from one disaggregated embryo separately into a well of 4-well dish coated with 0.1% gelatin and covered with MEF in standard TS medium including F4H. On day 3, XEN-like cells colonies appeared. We picked these colonies, disaggregated them by a glass pipette or by TrypLE Express for 5 min at 37 °C, and passaged them into a well of a 4-well dish. (We did not succeed in deriving post-XEN cell lines from single GFP+ cells that we placed in wells of a 96-well dish.) We thus derived, using the disaggregation method, a total of 30 post-XEN cell lines from 30 E6.5 embryos, at a 100% success rate (Table 1).

As was the case with our pre-XEN cell lines and post-XEN cell lines derived from E6.5 whole embryos, cells are immunoreactive for XEN markers GATA4, GATA6, SOX7, SOX17, and DAB2, but negative for ES cell markers OCT4 and NANOG, and negative for TS cell marker CDX2 (Fig. 3b). Thus, we increased the success rate of post-XEN cell derivation from 79% for whole E6.5 embryos to 100% for disaggregated E6.5 embryos.

### Derivation of post-XEN cell lines from E5.5 whole embryos

**Figure 4:**
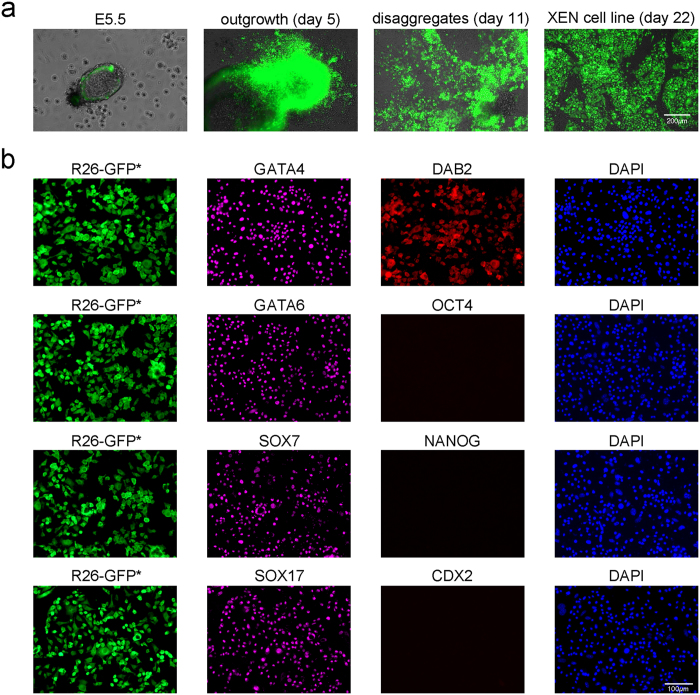
Derivation of post-XEN cell lines from whole E5.5 embryos. (**a**) Sox17-Cre × R26-tauGFP41 E5.5 embryo and post-XEN cell line. From left to right: whole E5.5 embryo, large outgrowth of embryo after 5 days in culture, cells after outgrowth disaggregation and culture for 11 days, and established post-XEN cell line X-E5.5-8 on day 22. Combined intrinsic green fluorescence of GFP/bright-field image. (**b**) Fluorescence analysis of post-XEN cell line X-E5.5-10. First column: intrinsic (indicated with an asterisk after GFP) green fluorescence of GFP from the *ROSA26* locus after activation by Cre recombinase that is expressed from the gene-targeted *Sox17* locus. Second column: cells are immunoreactive (magenta) for XEN markers GATA4, GATA6, SOX7, and SOX17. Third column: cells are stained for XEN marker DAB2, but not for ES cell markers OCT4 and NANOG, and not for TS cell marker CDX2. Fourth column: DAPI (blue).

In our final set of experiments, we derived post-XEN cell lines from 5.5 day-old embryos. We isolated 17 embryos from 33 E5.5 implantation sites from a mating of three homozygous R26-tauGFP41 females with three heterozygous Sox17-Cre males, and a mating of a homozygous ROSA-STOP-taulacZ female with a heterozygous Sox17-Cre male (Table 1). We removed as much of the ectoplacental cone as possible, and transferred one whole embryo separately into a well of a 4-well dish coated with 0.1% gelatin and covered with MEF, in TS medium including F4H (Fig. 4a). In our experience, the less ectoplacental cone and extraembryonic ectoderm remains in a dissected embryo, the easier it is to derive a post-XEN cell line. We surmise that ectoplacental cone cells and extraembryonic ectoderm cells (trophoblast-derived cells) compete with or inhibit XEN cells in culture. For 14/17 embryos, large outgrowths had appeared by day 5. We disaggregated these outgrowths with TrypLE Express on day 7, and passaged the cells into a well of a 4-well dish. On day 11, XEN-like colonies had accumulated. We picked these colonies, combined them, disaggregated them with a glass pipette or with TrpLE Express for 5 min at 37 °C, and passaged them into a well of a 4-well dish. We thus established 14 post-XEN cell lines after ~21 days in culture. For the remaining 3/17 embryos, trophoblast-derived cells surrounded the outgrowth. We performed a disaggregation by pipette on day 3, and switched to ES medium with LIF. When XEN cells became abundant, we passaged the cells onto gelatin-coated dishes without MEF, and switched back to TS medium including F4H. Three post-XEN cell lines were established after ~50 days culture. We thus derived a total of 17 post-XEN cell lines from 17 E5.5 embryos, at a 100% success rate (Table 1).

These post-XEN cell lines are immunoreactive for XEN cell markers GATA4, GATA6, SOX7, SOX17, and DAB2, but negative for ES cell markers OCT4 and NANOG, and negative for TS cell marker CDX2 (Fig. 4b).

### Post-XEN cell morphology, population characteristics, and *in vitro* differentiation

**Figure 5:**
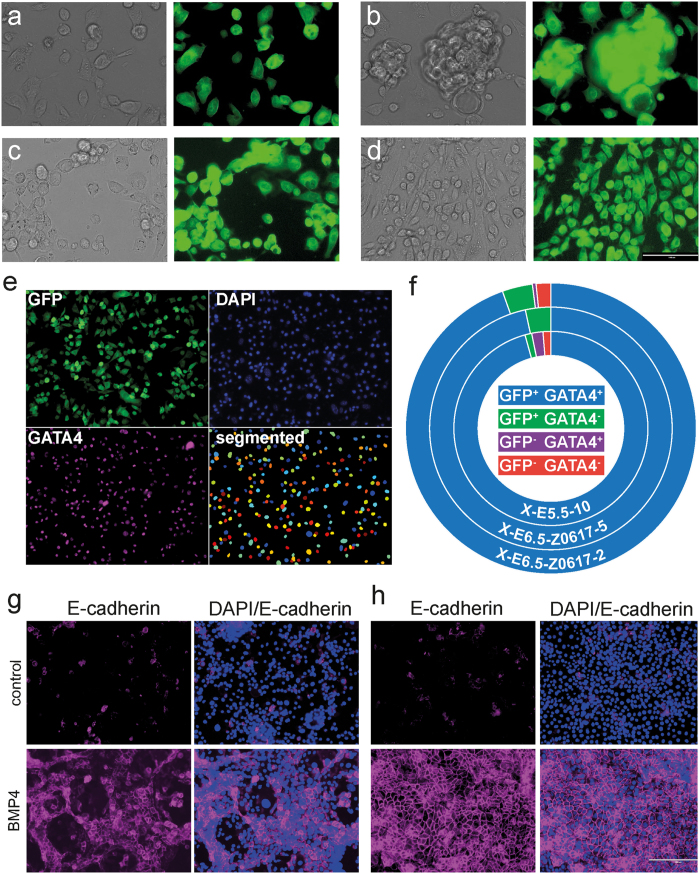
Post-XEN cell morphology in culture, population characteristics, and *in vitro* differentiation with BMP4. (**a**) Post-XEN cells X-E6.5-Z0617-5 cultured at low density contain a refractile, round cell type, and an epithelioid cell type. (**b**) Post-XEN cells X-E6.5-Z0617-5 cultured to near-confluency show the presence of individual cells and an epithelial sheet of cells. (**c**) Post-XEN cells X-E6.5-Z0617-5 participate in a lattice-type structure. (**d**) A subcloned cell line of X-E6.5-Z0617-5 continues to contain a refractile, round cell type, and an epithelioid cell type. (**e**) Analysis of population characteristics of the post-XEN cell lines X-E5.5-10, X-E6.5-Z0617-5, and X-E6.5-Z0617-2. Representative images of fields of view used to analyze Z0617-5 are shown. The GFP signal reflects intrinsic fluorescence, the GATA4 signal reflects immunoreactivity, and DAPI was used to counterstain the nuclei. The bottom right image shows the segmented nuclei of cells selected for marker analysis after the DAPI signal had undergone thresholding, declumping, and filtering for size and nuclear roundness. The individual cells are shown in multiple colors to clearly show the distinct cells that were used for analysis. Three sets of images were evaluated per cell line, and 500–1000 cells were evaluated per cell line. (**f**) Marker characteristics of the three cell lines are shown in the form of a doughnut chart, as determined by intrinsic fluorescence of GFP and immunoreactivity for GATA4. Cells are divided into four populations. (**g,h**) Immunofluorescence analysis of pre-XEN cell line X47 (**g**) and post-XEN cell line X-E6.5-Z0617-5 (**h**). Expression of the VE marker E-cadherin is higher after treatment with 10 ng/ml BMP4, in gelatin-coated plates. Left columns: E-cadherin (magenta). Right columns: DAPI signal (blue) merged with E-cadherin (magenta).

Like pre-XEN cell lines^10^, our cultures of post-XEN cell lines contain at least two cell morphologies: a rounded, highly refractile cell type and a more stellate epithelial-like cell type (Fig. 5a). At higher densities, post-XEN cells can form epithelial sheets (Fig. 5b) and often a lattice-type structure (Fig. 5c). To determine if these two cell morphologies represent two cell types in the cultures, we FACS-sorted single GFP+ post-XEN cells (X-E6.5-Z0617-5) directly into wells of a 96-well dish, and derived two subclones. Cells of these subclones continued to exhibit either round or epithelial-like cell morphologies (Fig. 5d).

To evaluate the population characteristics of post-XEN cell lines, we analyzed three GFP-expressing cell lines (X-E6.5-Z0617-5, X-E6.5-Z0617-2 and X-E5.5-10) by immunofluorescence for GATA4 and counterstaining with DAPI. Sets of fluorescence images were captured for each line visualizing the intrinsic fluorescence of GFP, DAPI, and GATA4 immunoreactivity (Fig. 5e). The images were evaluated with a custom CellProfiler pipeline that segmented cells using the DAPI fluorescent signal. A cutoff for size and nuclear roundness was used to exclude the MEF population as much as possible. We then evaluated each cell for levels of GFP and GATA4 signal. In these three post-XEN cell lines, >94% of cells evaluated exhibited GFP fluorescence and are GATA4+ (Fig. 5f). The populations of cells that expressed either GFP or GATA4 but not both represented 0–3% of the cells evaluated. The remaining population of cells, which expressed neither GFP nor GATA4, may reflect another cell type, or MEFs that escaped exclusion during the cell identification step in the custom CellProfiler pipeline.

We asked if post-XEN cells can differentiate into a VE identity by incubation with BMP4^38^,^39^. We cultured four post-XEN cell lines (X-E5.5-9, X-E6.5-Z0617-2, X-E6.5-Z0617-5 and X-E6.5-78097-7) and three pre-XEN cell lines (X42, X47, X-ICM-4) in gelatin-coated dishes in TS medium with F4H, plus 10 ng/ml BMP4. In parallel, we cultured the same cell lines in TS medium with F4H without BMP4. Four days later, we performed immunofluorescence for E-cadherin, a VE marker. We found that culture with BMP4 induces expression of E-cadherin in post-XEN cells, as well as in pre-XEN cells^10^ (Fig. 5g-h).

### NanoString gene expression analyses of XEN and ES cell lines

**Figure 6:**
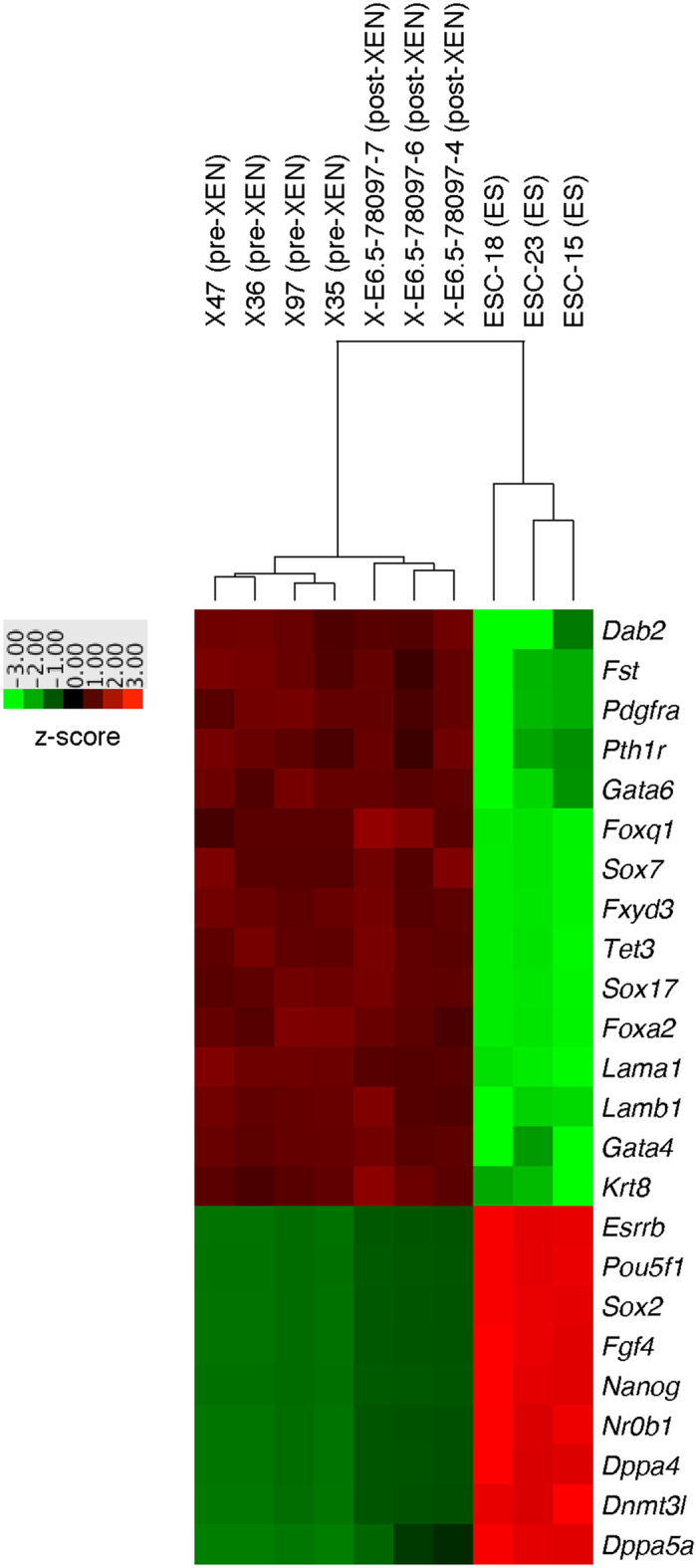
NanoString gene expression analyses of XEN and ES cell lines. Heatmap analysis of four pre-XEN cell lines, three post-XEN cell lines, and three ES cell lines. Red means high expression level, green means lowly expression level. Agglomerative clustering was performed with nSolver software. Among the classical XEN cell markers are *Dab2, Pdgfra2, Gata6, Sox7, Sox17,* and *Gata4.* Among the classical ES cell markers are *Oct4/Pou5f1, Sox2, Ffg4,* and *Nanog*. Pre-XEN and post-XEN cell lines share a gene expression profile that is typical for XEN cell lines.

We applied the NanoString multiplex platform for gene expression^40^,^41^,^42^ and agglomerative clustering, in order to compare the patterns of expression of selected genes in four pre-XEN cell lines, three post-XEN cell lines, and three ES cell lines that we had derived in other experiments (Fig. 6). We find that all our XEN cell lines have high levels of expression of XEN-specific genes, such as *Dab2, Gata4, Gata6, Pdgfra, Sox7,* and *Sox17,* versus low or no expression of ES cell-specific genes such as *Nanog, Pou5f1/Oct4, Sox2,* and *Nr0b1*. There is no expression of EpiSC-specific genes such as *Cer1* and *Fgf5* (data not shown). Thus, the NanoString gene expression analysis confirms and extends the immunofluorescence profiles.

### Post-XEN cells contribute to the extraembryonic endoderm of chimeras after injection into blastocyts

A final and stringent test of the potency of stem cells is to assess their contribution to embryonic and extraembryonic tissues *in vivo*. We injected into blastocysts (B6D2F2, C57BL/6 J, or CD1) cells of two R26-tauGFP41+ pre-XEN cell lines (X-ICM-4 and X-ICM-5) and one PDGFRa-GFP+ pre-XEN cell line (X47) that we had derived from isolated ICMs or blastocysts, and cells from four R26-tauGFP41+ post-XEN cell lines (X-E6.5-Z0617-2, X-E6.5-Z0617-5, X-E5.5-6 and X-E5.5-9) and one PDGFRa-GFP+ post-XEN cell line (X-E6.5-Z0663-9) that we had derived from E5.5 embryos or disaggregated E6.5 embryos (Table 2). For pre-XEN cells, we identified 3 chimeras among 8 E6.5 embryos (38%), and 15 chimeras among 43 E7.5 embryos (35%), from a total of 82 injected blastocysts (18/82 = 22%). For post-XEN cells, we identified 19 chimeras among 49 E6.5 embryos (39%) and 30 chimeras among 83 E7.5 embryos (36%), from a total of 196 injected blastocysts (49/196 = 25%). The percentages of identifiable chimeras among embryos and with regard to injected blastocysts are thus comparable for pre-XEN and post-XEN cell lines.

**Figure 7:**
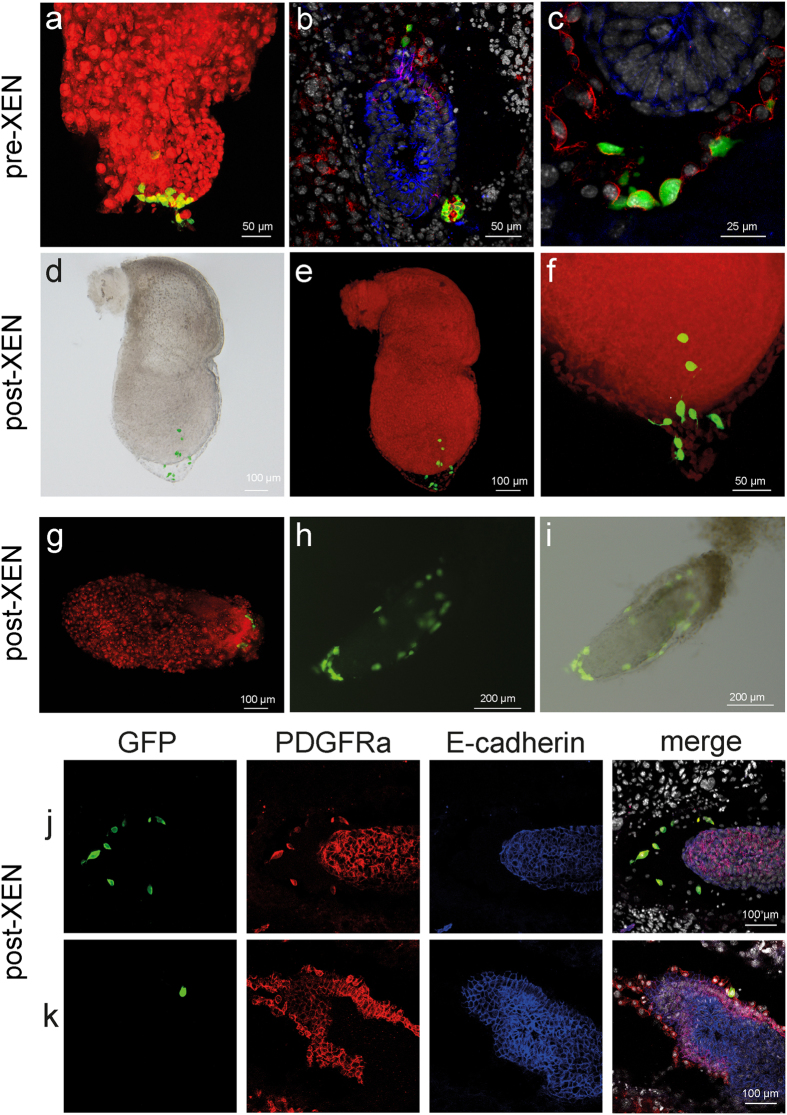
XEN cells contribute to the parietal endoderm of chimeric embryos. (**a–c**) Pre-XEN cell line X-ICM-4 contributes to the PE of E6.5 and E7.5 embryos. GFP+ cells are derived from the injected pre-XEN cells. (**a**) Wholemount of E7.5 embryo. (**b,c**) Sections of the decidua of E6.5 embryos, showing the merged image of immunofluorescence for PDGFRa (red) and E-cadherin (blue), and fluorescence of DAPI (white) and GFP (green). (**d–k**) Post-XEN cell lines (X-E5.5-9, X-E6.5-Z0617-5, and X-E6.5-Z0617-2) contribute to the PE of E6.5 or E7.5 embryos. (**d–f**) Wholemount. GFP+ cells derived from X-E5.5-9 contribute to an E7.5 embryo. (**g**) Wholemount. GFP+ cells derived from X-E6.5-Z0617-5 contribute to an E7.5 embryo. (**h,i**) Wholemount. GFP+ cells derived from X-E6.5-Z0617-2 contribute to an E6.5 embryo. (**j**,**k**) Sections of the decidua of E7.5 embryos containing GFP+ cells derived from X-E6.5-Z0617-5, with immunofluorescence for PDGFRa (red) and E-cadherin (blue), fluorescence of DAPI (white) and GFP (green), and merge. The single GFP+ cell in (**k**) apposes but does not belong to the VE, as it is not immunoreactive for E-cadherin. (**a–g**) and (**j,k**) were imaged using a Zeiss LSM 710 confocal microscope, and (**h–i**) were imaged using a Nikon SMZ25 stereofluorescence microscope. In (**a**) and (**e–g**) the red counterstain is propidium iodide.

We found that pre-XEN and post-XEN cells contribute to extraembryonic endoderm at E6.5 or E7.5 (Fig. 7), as is typical for XEN cells injected into blastocysts^10^,^20^. We sectioned the decidua of E6.5 or E7.5 embryos obtained by injecting pre-XEN or post-XEN cells into blastocysts, and performed immunofluorescence with antibodies against PDGFRa and E-cadherin. PDGFRa is expressed in the PE and VE^32^, and E-cadherin is expressed in the VE and epiblast^16^,^43^,^44^. Therefore, extraembryonic endoderm cells that are only immunoreactive for PDGFRa are PE, and cells that are immunoreactive for both PDGFRa and E-cadherin are VE. We analyzed 103 GFP+ cells in E6.5 chimeras obtained by injection of X-ICM-4 (pre-XEN), and 123 GFP+ cells in E7.5 chimeras with X-ICM-5 (pre-XEN), and found that all 226 GFP+ cells reside within the PE region and are immunoreactive for PDGFRa but not for E-cadherin; thus, no GFP+ cell contributed to the VE (Fig. 7a–c). We analyzed 75 GFP+ cells in E6.5 chimeras and 142 GFP+ cells in E7.5 chimeras obtained by injection of X-E6.5-Z0617-5 (post-XEN), and found that all 217 GFP+ cells reside within the PE region and are immunoreactive for PDGFRa but not for E-cadherin; thus, no GFP+ cell contributed to the VE (Fig. 7d–k).

## Discussion

We report here that XEN cell lines can be derived efficiently from postimplantation embryos, and from a wide variety of strains and crosses. We believe that it is prudent to continue to refer to these two types of cell lines with regard to the embryonic stage from which they were derived. Additional work will be necessary to identify and characterize possible differences, subtle or substantial, between pre-XEN and post-XEN cell lines.

With our E5.5 whole-embryo method and E6.5 disaggregated-embryo method, we attained a success rate of 100%: we derived 17 post-XEN cell lines from 17 embryos and 30 post-XEN cell lines from 30 embryos, respectively. There are only ~11 PrE cells at the late blastocyst stage^45^. As E5.5 and E6.5 embryos contain ~95 and ~250 extraembryonic endoderm cells respectively^46^, these higher numbers of potential source cells may explain the 100% efficiency of XEN cell line derivation from postimplantation embryos. Other, experimental, reasons for the highly efficient derivation may be the reduction of the negative effects of trophoblast-derived cells on XEN cells in the disaggregated E.6 embryo method, and the promotion of XEN cells over trophoblast-derived cells by culturing in ES medium with LIF in the whole-embryo E5.5 embryo method. Regardless of these numerical and experimental explanations, a more exciting, and biological, explanation is that extraembryonic tissues preserve developmental plasticity through implantation.

Since the first report of XEN cell derivation from blastocysts in 2005 (ref. 10), there are three types of methods in place to derive XEN cell lines, either directly from blastocysts^10^,^20^ or by conversion from ES cells^20^,^21^,^22^,^23^,^24^,^25^,^26^ or, recently, by reprogramming from fibroblasts^27^. The success rate for derivation of XEN cell lines from blastocysts was 21% in TS medium including F4H and 56% in ES medium supplemented with LIF^20^, which is identical to our success rate of 57% in ES medium supplemented with LIF. Recently, LIF has been reported to support PrE expansion during pre-implantation embryo development^47^, and LIF could also be supporting XEN cell expansion. The conventional protocol to derive ES cell lines from blastocysts involves also ES medium supplemented with LIF^48^,^49^; this medium can be used to derive XEN cell lines from blastocysts, by picking XEN cell colonies or by removing ES cell colonies, but there is always the risk of deriving ES cell lines^50^. The indirect generation of XEN cell lines from ES cell lines requires, obviously, the prior generation or availability of such lines.

TS cell lines can be derived from the extraembryonic ectoderm of postimplantation embryos in TS medium including FGF4 and heparin^9^,^11^. Interestingly, we did not derive TS cell lines from either whole or disaggregated embryos using this medium. We did observe transiently cells and colonies with a morphology that is consistent with TS cells or EpiSCs, but they disappeared from the culture with time. In our immunofluorescence analyses, our XEN cell lines do not contain cells that express the trophectoderm marker Cdx2, and our Nanostring analyses do not reveal expression of TS-specific genes either. The cell lines that we derived from postimplantation embryos in TS medium are thus not TS cell lines, not ES cell lines, and not EpiSC lines, but represent XEN cell lines. Why then are XEN cell lines obtained rather than TS cell lines with our method, which cultures the cells in the same TS medium? The protocol for deriving TS cell lines from postimplantation embryos prescribes that the extraembryonic ectoderm be dissected out and that the embryo and the VE and PE be discarded^12^. By contrast, we remove the ectoplacental cone from the embryo. We speculate that the VE and PE surrounding the extraembryonic ectoderm inhibit TS cell growth, and/or that VE- and PE-derived cells divide at a faster rate than TS cells and dominate with time the cell culture.

Chemical reprogramming of mouse fibroblasts to the iPS cells involves a XEN-like intermediary stage as a bridge between somatic and pluripotent cells^19^. In another study, iXEN clones arise in parallel to iPS clones during OSKM-mediated reprogramming of mouse fibroblasts^27^. More knowledge about the derivation, biology, culture, and conversion of XEN cell lines may be beneficial to develop robust and efficient protocols for the derivation of iPS cell lines by chemical reprogramming with small molecules.

In future experiments, it will be interesting to determine the cell type(s) that are at the origin of the post-XEN cell lines.

## Methods

### Mouse strains

The PDGFRa-GFP strain^28^ was obtained from The Jackson Laboratory (JAX), strain 7669, strain name B6.129S4-Pdgfra<tm11(EGFP)>Sor/J. The CAG::mRFP1 strain^29^ was obtained from JAX, strain 5645, strain name Tg(CAG-mRFP1)1F1Hadj/J. The D4/XEGFP strain^30^ was obtained from JAX, strain 3116, strain name Tg(CAG-EGFP)D4Nagy/J. The Sox17-Cre strain^34^ was obtained from MMRRC, strain 036463-UNC, strain name Sox17<tm2(EGFP/cre)Mgn>/Mmnc. (Although this strain is reported to contain and express GFP, and we confirm the presence of GFP in this targeted insertion in the *Sox17* locus, we cannot detect GFP expression in embryos and cell lines derived from them.) The Xist1loxGFP strain^35^ was provided by the RIKEN BioResource Center through the National BioResource Project of the MEXT (Japan), strain RBRC01260, strain name B6;129(Cg)-Xist<tm2Sado>. The GATA6-mTomato strain^36^ was provided by the RIKEN BioResource Center, strain RBRC04900, strain name B6;B6C3(129)-Tg(GATA6-mTomato)3Hmd. The Cdx2-GFP strain^37^ was obtained from JAX, strain 18983, strain name Cdx2<tm1Yxz>/J. The ROSA26-STOP-taulacZ reporter strain^51^ was generated by Dr. Ivan Rodriguez in the laboratory of P.M. at The Rockefeller University, and is publicly available from JAX, strain 6744, STOCK Gt(ROSA)26Sor<tm1.1Mom>/MomJ. The R26-tauGFP41 reporter strain^52^ was a gift from Dr. Uli Boehm, Universität des Saarlandes, Homburg, Germany. For the preparation of mouse embryonic fibroblasts, strain 3208, strain name Tg(DR4)1Jae was obtained from JAX.

### TS cell medium

Advanced RPMI-1640 (Gibco, #12633-012) was supplemented with 20% (vol/vol) FBS (HyClone, #SH30071.03), 2 mM GlutaMAX Supplement (Gibco #35050), 1% penicillin/streptomycin (Specialty Media #TMS-AB2-C), 0.1 mM β-mercaptoethanol (Gibco #21985-023), 1 mM sodium pyruvate (Gibco #11360-039), supplemented with 25 ng/ml FGF4 (Peprotech #100-31) and 1 μg/ml heparin (Sigma #H3149).

### ES cell medium

ES cell lines were maintained on MEF-coated, pregelatinized tissue culture dishes (Falcon) in DMEM (Specialty Media #SLM-220) supplemented with 15% FBS (HyClone #SH30071.03), 2 mM GlutaMAX Supplement, 1% penicillin/streptomycin, 1% β-mercaptoethanol (Specialty Media #ES-007-E), 0.1 mM nonessential amino acids (Gibco #11140-035), 1 mM sodium pyruvate, and 1000 IU/ml leukemia inhibitory factor (LIF) (Millipore #ESG1107).

### Derivation of ES cell lines

Embryos were collected at the 2–8 cell stage by flushing oviducts using M2 medium (Sigma #M7167), and cultured in KSOM medium (Millipore #MR-106-D) to the blastocyst stage. The zona pellucida of blastocysts was then removed using acid Tyrode solution (Sigma #T1788). Blastocysts were transferred separately into a well of a 96-well dish (Falcon) coated with 0.1% gelatin (Specialty Media #ES-006-B) and covered with MEF in ES medium supplemented with LIF and 1 μM PD0325901 (Axon #1408) and 3 μM CHIR99021 (Axon #1386), a combination of chemicals that is typically referred to as “2i”. After 4 days TrypLE Express (Gibco #12604-013) was used to disaggregate the embryonic outgrowths, and cells were passaged to a well of 24-well dish to derive ES cell lines.

### Derivation of pre-XEN cell lines from blastocysts

Embryos were collected at the 2–8 cell stage embryos and cultured in in KSOM medium to the blastocyst stage. The zona pellucida of blastocysts was removed using acid Tyrode solution. Blastocysts were transferred separately into a well of a 4 well-dish (Nunc #176740) coated with 0.1% gelatin and covered with MEF in ES medium supplemented with LIF. The XEN lines were derived as described^20^.

### Derivation of post-XEN cell lines from whole E5.5 or E6.5 embryos

The ectoplacental cone was removed with forceps or needles. A whole embryo was placed in a well of a 4-well dish (Nunc #176740) coated with 0.1% gelatin and covered with MEF in TS medium including F4H. After the embryos formed a large outgrowth, TrypLE Express was used to disaggregate the outgrowths and passage cells into a 4-well dish. When cells reached 70–80% confluency, they were passaged into a well of a 12-well dish until XEN cell lines were obtained, which were then passaged into a well of a 6-well dish. If the outgrowth of an E5.5 embryo grew well and XEN cells thrived, we continued to culture cells in TS medium including F4H. But if the outgrowth grew slowly and XEN cells were surrounded by trophoblast-derived cells, we cultured cells in ES medium supplemented with LIF, culture conditions that would inhibit trophoblast-derived cells; when XEN cells started to become abundant, we switched to TS medium including F4H. To prepare XEN cells for RNA extraction, cells were cultured in dishes coated with 0.1% gelatin but without MEF in TS medium including F4H.

### Derivation of post-XEN cell lines at E6.5 from disaggregated embryos

The ectoplacental cone was removed with forceps or needles. A whole embryo was treated with 0.1 mg/ml collagenase (Gibco #17104-019) and 0.01 mg/ml deoxyribonuclease (Gibco #D5025) for 20–30 min at room temperature, followed by 0.2 mg/ml TrypLE for 5 min at room temperature. The embryo was disaggregated into single cells using a glass pipette with a diameter of 50–60 μm. Cells were transferred into a well of a 4-well dish (Nunc #176740) coated with 0.1% gelatin and covered with MEF in TS medium including F4H. Three days later XEN colonies appeared. We picked these colonies, disaggregated them with a glass pipette or by TrypLE Express for 5 min at 37 °C, and passaged them into a well of a 4-well dish. When cells reached 70–80% confluency, they were passaged into a well of a 12-well dish until XEN cell lines were obtained, which were then passaged into a well of a 6-well dish.

### Population characteristics

Images representing Fields of View of post-XEN cell lines were imaged for intrinsic fluorescence of GFP, fluorescence of DAPI, and GATA4 immunoreactivity. Automated cell population characteristics were determined with CellProfiler http://cellprofiler.org/ (Broad Institute, Cambridge, MA, USA). The DAPI signal was used to segment individual cells by thresholding, de-clumping, and applying size and roundness filter in an effort to evaluated single cells that are not MEFs. After filtering, the GFP and GATA4 signals were evaluated in each cell, and the population characteristics for these two markers were quantified.

### *In vitro* differentiation

Gelatin-coated plates were prepared by coating with 0.1% gelatin overnight at room temperature. XEN cells were cultured in TS medium with F4H and with or without 10 ng/mL BMP4 (Peprotech, 120–05) on gelatin-coated plates for four days.

### Immunofluorescence and imaging

Cell lines X42, X44, X47 (PDGFRa-GFP × CAG::mRFP1); X35, X36 (B2D6F1 × D4/EGFP); X97, X107 (D4/EGFP × DBA2/N); X-E6.5-81346-8 (ROSA-STOP-taulacZ x Sox17-Cre); X-E6.5-Z0617-2, X-E6.5-Z0617-5, X-E6.5-Z0617-8 (R26-tauGFP41 x Sox17-Cre); X-E6.5-78097-4 (Xist1loxGFP × DBA/2 N); X-E6.5-82278-4 (Gata6-mTomato × Cdx2-GFP); X-E6.5-Z0663-12 (CD1 × PDGFRa-GFP); and X-E5.5-6, X-E5.5-8, X-E5.5-9, X-E5.5-10, X-E5.5-13 (R26-tauGFP41 × Sox17-Cre) were cultured in 4-well or 24-well dishes. Cells were fixed in 4% paraformaldehyde at 4 °C overnight or room temperature for 30 min, permeabilized with 0.1% Triton X-100 in 1× PBS (1× PBST) for 30 min and blocked with 5% normal donkey serum (Jackson ImmunoResearch Laboratories, #017-000-121) diluted in 1× PBST (blocking solution) for 1 hr. Primary antibodies were diluted at 1:50–1:200 in blocking solution and samples incubated at 4 °C rotating overnight. After three 10-min washes in 1× PBST 10 min, samples were incubated for 1–1.5 hr at room temperature in a 1:500 dilution of secondary antibody in blocking solution, then washed and covered with 1× PBST containing DAPI. Images were taken with an AMG EVOS (Life Technologies), Zeiss LSM 710 confocal microscope, and Nikon SMZ25 stereofluorescence microscope.

Primary antibodies from Santa Cruz Biotechnology were against GATA4 (#SC-1237), DAB2 (#SC-13982), OCT3-4 (#SC-5279), NANOG (#SC-376915), and CDX2 (#SC-166830). Primary antibodies from R&D Systems were against GATA6 (#AF1700), SOX7 (#AF2766), SOX17 (#AF1924), and PDGFRa (#AF1062). Primary antibodies against E-cadherin (ECCD2) were from Invitrogen (#13-1900). Secondary antibodies from Jackson ImmunoResearch Laboratories were Cy5™ AffiniPure Donkey anti-goat IgG (H+L) (#705-175-147), and Cy5™ AffiniPure Donkey anti-rabbit IgG (H+L) (#711-175-152). Secondary antibodies from Invitrogen were Donkey anti-rabbit IgG (H+L) with Alexa Fluor 546 (#A10040), Goat anti-rat IgG (H+L) with Alexa Fluor 546 (#A11081), Donkey anti-mouse IgG with Alexa Fluor 546 (#A10036), Donkey anti-mouse IgG with Alexa Fluor 488 (#A21202), and Goat anti-chicken IgY (H+L) with Alexa Fluor 488 (#A11039).

### NanoString multiplex gene expression analysis

Cells were cultured in 12-well plates with TS medium including F4H or ES medium supplemented with LIF. Dissociated cells were collected by trypsinization and centrifugation. Cell pellets were dispensed directly in RNAlater Stabilization Solution (Qiagen) and stored in −80 °C for later use. Cell pellets were lysed in RLT Lysis Plus Buffer using a TissueLyser LT (Qiagen) at 40 Hz for 2 min. Total RNA extraction was performed using RNeasy Plus Micro kit (Qiagen) according to manufacturer’s protocol. The custom NanoString CodeSet “Extra” was used. 100 ng of total RNA samples were hybridized at 65 °C for 18 hr and processed with the nCounter Analysis System GEN1 (NanoString Technologies). The reporter counts were processed using nSolver Analysis Software v2.5 (NanoString). Two normalizations were performed to the counts, the first normalization to the generic positive controls, followed by normalization to the reference genes, *Actb* and *Gapdh*. Normalized counts are displayed in a heatmap generated by the nSolver Analysis Software v2.5, using agglomerative clustering.

### NanoString probe sequences

Below are the nucleotide sequences of the NanoString probes; first line is the capture probe, second line the reporter probe.

*Actb*

*ACGATGGAGGGGCCGGACTCATCGTACTCCTGCTTGC*

*GGGTGTAAAACGCAGCTCAGTAACAGTCCGCCTAGAAGCACTTGCGGTGC*

*Dab2*

*GTCTCCTCGAGCATCAGGCACATCATCAATACCGATTAGCTTGGC*

*CCAGCTGCTGCCATTCCCTTGAGTTTCATCATAGAATCCTGACTCATTTT*

*Dnmt3l*

*GAGGCAGCGCATACTGCAGGATCCGGTGGAACTGGAACATG*

*CCATGAATATCCAGAAGAAGGGCCGCTGACTCTCCTGGC*

*Dppa4*

*CAAGTCTTTACAGTTGACTGCTGAACTGGTTATGACGCCCGTTGTGCTGG*

*CACTACAACCCAGGGAAGAGGACATGCATGCGGAGGCTACAGGTATAAGC*

*Dppa5a*

*CGCACGGCCCACAGCTCCAGGTTCAGGAAGTTTTAGTAC*

*CCTGCCAAGGAACCAGACTTCAGGGAAGACGAGATCAAGCTTATCCACCA*

*Esrrb*

*TTTCCAGAATGAACCGCTTCATCTTTAGGACATCCTGTCAACCCAAACCC*

*GAGCAGGTAAAGCCGGAGGACTTGTCATGAAAGTGGCGTGTCCAT*

*Fgf4*

*ATTCTGGTAACAAAATTCCAAAGATACAGTCTTGTCCCTGGGCGCAGGAA*

*ACAGACCGACTCGGTAACAGTGGCAGATACAGAGCAGAAACATCAAACCC*

*Foxa2*

*CACAGACAGGTGAGACTGCTCCCTTGAGGCCTGAAG*

*TCCCTTCCCTATTTAGAATGACAGATCACTGTGGCCCATCTATTTAGGGA*

*Foxq1*

*GCTGTCCTTACTCCGAGGTTTAGAGACTTTGAGCGGAAGACAAGCG*

*TTTTGATTGTTGGGTGAAGTGAGGAGTGGAGTGATAGAAGTTGGTGCAGT*

*Fst*

*AGGACTTTGTGATACACTTTCCCTCATAGGCTAATCCAATGGATCTGCCC*

*CTTGGAATCCCATAGGCATTTTTTCCCGCCGCCACACTGGATATCTTCAC*

*Fxyd3*

*TGAGCCCGCCGACTCGGAGGCTGTACCAATCATAGTAGAAAGGATCATTT*

*GCCACTCATAAGGACTATAATGCCCAGGGCACAGAGAATCCCTGCACAAA*

*Gapdh*

*ATACTTGGCAGGTTTCTCCAGGCGGCACGTCAGATCCACGACG*

*CCTCAGATGCCTGCTTCACCACCTTCTTGATGTCATC*

*Gata4*

*AGAGCCAGGTAACTGTCTGACTTAAGAGGGCTTGGCTTGG*

*GCAAACAGTCTGTATTTTCTAGACAAAGGATCTGTGCTGGAGAAAGTCCC*

*Gata6*

*ATCTGGACTGCTGGACAATATCAGACACAAGTGGTATGAGGCCTTCAGAG*

*GGCACAGAAATCACGCATCGAAGGAATGTTATGTCTGCATTTTTGCTGCC*

*Krt8*

*TTCCCATCTCGGGTTTCAATCTTCTTCACAACCACAGCCTTG*

*CAGTGGCCATTCACTTGGACACGACATCAGAAGACTCGGACACCAGC*

*Lama1*

*CATTGGCTAAATCGGCATGGCGGTCATCCTTGATACAGACAGAACTCAGA*

*CATAACCTTTCCTACATGGACACTGACCTGGCCACTTTC*

*Lamb1*

*CCAGGAAGGAATGCGGTCCTGAATGTACTGCCTTTCCACCACAATGAC*

*AAAAACTCCAAATAAGCCCCTTCAGGCACCCGGACGAACCCAGGTCCTGT*

*Nanog*

*CATATTTCACCTGGTGGAGTCACAGAGTAGTTCAGGAATAATTCCAAGGC*

*GAAGGAACCTGGCTTTGCCCTGACTTTAAGCCCAGATGTTGCGTAAGTCT*

*Nr0b1*

*CACTTGAAAAAGAAACTCTTGATGGCCTGGACCGCAGCAGCTGGGAGCAA*

*CCTTTCAGATAGGCATACTCTTTGGTGTCAATGTTCAGACTCCAG*

*Pdgfra*

*TATGGAGTAAGTCGCTCTCACACACTTACCACACCACCATGTTGGGAACA*

*AAACATGAACAGGGGTATCTGGAAGCCATCTTGTATTGGAAGACCCTTCC*

*Pou5f1*

*ACATGGTCTCCAGACTCCACCTCACACGGTTCTCAATG*

*AGTGATCTGCTGTAGGGAGGGCTTCGGGCACTTCAGAA*

*Pth1r*

*CCTGGGCACGGTGCAGCAGGAAAATCTGTTCCTCTTTGGTAAAGACATCG*

*TGCTGTGTGCAGAACTTCCTTGAGCAGCTTGTCACATTGCG*

*Sox2*

*CCCCGCCGCCCTCAGGTTTTCTCTGTACAAAAATAGTCCCCCAAAAAGAA*

*TGCGTAGTTTTTTTCCTCCAGATCTATACATGGTCCGATTCCCCCGCCCT*

*Sox7*

*AGAAATCAGCACACCCCAACACTTTTGTGGACAGACGTTTTAGGTTTCTA*

*TCATCTTTTGCATCTGTAGATAAGAGTATGCTACAGCTCTGCTCT*

*Sox17*

*GGCAGATACTGTTCGAATTCCGTGCGGTCCACCTC*

*TGTCCCTGGTAGGGAAGACCCATCTCGGGCTTATACACAAAG*

*Tet3*

*TTTTCAAAGAGCTGAATGAATGCACCAGGATTTTAGGATGGGCGTGTTTC*

*GTCAGGCCCAACCTCTCAATGTCACAGAAATTAAAGCACCAACGTTCTCA*

### Ethics statement

All animal studies were carried out in accordance with the German Animal Welfare Act, European Communities Council Directive 2010/63/EU, and institutional ethical and animal welfare guidelines of the Max Planck Institute of Biophysics and the Max Planck Research Unit for Neurogenetics. All experimental protocols were approved by the Regierungspräsidium Darmstadt and the Veterinäramt of the City of Frankfurt.

## Additional Information

**How to cite this article**: Lin, J. *et al*. Efficient derivation of extraembryonic endoderm stem cell lines from mouse postimplantation embryos. *Sci. Rep.* **6**, 39457; doi: 10.1038/srep39457 (2016).

**Publisher's note:** Springer Nature remains neutral with regard to jurisdictional claims in published maps and institutional affiliations.

## Figures and Tables

**Table 1:** Derivation of pre-XEN and post-XEN cell lines.

Method	Stage of embryo for derivation	Strain	No. embryos	No. XEN cell lines (%)	No. XEN cell lines heterozygous	No. XEN cell lines wild-type
Whole embryo	Blastocysts	B6D2F1 × D4/XEGFP	20	11 (55)	11	0
Whole embryo	Blastocysts	D4/XEGFP × DBA/2 N	20	11 (55)	11	0
Whole embryo	Blastocysts	PDGFRa-GFP× CAG::mRFP1	23	14 (61)	14	0
ICMs	Blastocysts	R26-tauGFP41 × Sox17-Cre	6	5 (83)	2	3
		Sum	69	41 (59)		
Whole embryo	E6.5	Xist1loxGFP ×DBA/2 N	19	15 (79)	5	10
Whole embryo	E6.5	Gata6-mTomato × Cdx2-GFP	6	6 (100)	3	3
Whole embryo	E6.5	ROSA-STOP-taulacZ × Sox17-Cre	13	9 (70)	1	8
		Sum	38	30 (79)		
Disaggregated embryo	E6.5	R26-tauGFP41 × Sox17-Cre	11	11 (100)	5	6
Disaggregated embryo	E6.5	CD1 × PDGFRa-GFP	12	12 (100)	5	7
Disaggregated embryo	E6.5	ROSA-STOP-taulacZ × Sox17-Cre	7	7 (100)	1	6
		Sum	30	30 (100)		
Whole embryo	E5.5	R26-tauGFP41 × Sox17-Cre	13	13 (100)	11	2
Whole embryo	E5.5	ROSA-STOP-taulacZ × Sox17-Cre	4	4 (100)	2	2
	E5.5	Sum	17	17 (100)		

**Table 2:** Post-XEN cell lines contribute to extraembryonic endoderm in chimeras.

Cell lines	Strains	No. Blastocysts injected	No. Blastocysts transferred	No. Implantation sites	No. E6.5 embryos	No. E6.5 chimeras	No. E7.5 embryos	No. E7.5 chimeras
Pre-XEN cells								
X-ICM-4	R26-tauGFP41 × Sox17-Cre	59	59	53	8	3	33	10
X-ICM-5	R26-tauGFP41 × Sox17-Cre	15	15	12			8	3
X47	PDGFRa-GFP× CAG::mRFP1	8	8	3			2	2
Sum		82	82	68	8	3	43	15
Post-XEN cells								
X-E6.5-Z0617-2	R26-tauGFP41 × Sox17-Cre	31	31	24	13	6	10	2
X-E6.5-Z0617-5	R26-tauGFP41 × Sox17-Cre	83	83	68	11	4	46	19
X-E6.5-Z0663-9	PDGFRa-GFP × CD1	15	15	10	4	1	5	1
X-E5.5-6	Sox17-Cre × R26-tauGFP41	22	22	16	13	6		
X-E5.5-9	Sox17-Cre × R26-tauGFP41	45	45	38	8	2	22	8
Sum		196	196	156	49	19	83	30
